# The chemokine receptor CX_3_CR1 is directly involved in the arrest of breast cancer cells to the skeleton

**DOI:** 10.1186/bcr3016

**Published:** 2011-09-20

**Authors:** Whitney L Jamieson-Gladney, Yun Zhang, Alan M Fong, Olimpia Meucci, Alessandro Fatatis

**Affiliations:** 1 Department of Pharmacology and Physiology, Drexel University College of Medicine, 245 N 15th Street, Philadelphia, PA 19102-1101, USA; 2 Department of Genetics and Tumor Cell Biology, St. Jude Children's Research Hospital, Memphis, TN 38105-3678,USA; 3 Thurston Arthritis Research Center, University of North Carolina at Chapel Hill, Chapel Hill, NC 27599-6011, USA; 4 Department of Pathology and Laboratory Medicine, Drexel University College of Medicine, 245 N 15th Street, Philadelphia, PA 19102-1101, USA

## Abstract

**Introduction:**

Skeletal metastases from breast adenocarcinoma are responsible for most of the morbidity and mortality associated with this tumor and represent a significant and unmet need for therapy. The arrival of circulating cancer cells to the skeleton depends first on the adhesive interactions with the endothelial cells lining the bone marrow sinusoids, and then the extravasation toward chemoattractant molecules produced by the surrounding bone stroma.

We have previously shown that the membrane-bound and cell-adhesive form of the chemokine fractalkine is exposed on the luminal side of human bone marrow endothelial cells and that bone stromal cells release the soluble and chemoattractant form of this chemokine. The goal of this study was to determine the role of fractalkine and its specific receptor CX_3_CR1 in the homing of circulating breast cancer cells to the skeleton.

**Methods:**

We employed a powerful pre-clinical animal model of hematogenous metastasis, in which fluorescent cancer cells are identified immediately after their arrival to the bone. We engineered cells to over-express either wild-type or functional mutants of CX_3_CR1 as well as employed transgenic mice knockout for fractalkine.

**Results:**

CX_3_CR1 protein is detected in human tissue microarrays of normal and malignant mammary glands. We also found that breast cancer cells expressing high levels of this receptor have a higher propensity to spread to the skeleton. Furthermore, studies with fractalkine-null transgenic mice indicate that the ablation of the adhesive and chemotactic ligand of CX_3_CR1 dramatically impairs the skeletal dissemination of circulating cancer cells. Finally, we conclusively confirmed the crucial role of CX_3_CR1 on breast cancer cells for both adhesion to bone marrow endothelium and extravasation into the bone stroma.

**Conclusions:**

We provide compelling evidence that the functional interactions between fractalkine produced by both the endothelial and stromal cells of bone marrow and the CX_3_CR1 receptor on breast cancer cells are determinant in the arrest and initial lodging needed for skeletal dissemination.

## Introduction

Currently, only six percent of women that are first diagnosed with breast adenocarcinoma present with metastases [1]. Unfortunately, between 20 and 50% of them will eventually develop a metastatic disease [1]. Metastases are responsible for an intolerably high number of deaths among patients that would otherwise be almost invariably cured by surgical resection and adjuvant therapy [2]. Autopsy studies have estimated that 70% of advanced breast cancer patients have skeletal metastases [3]. These secondary bone tumors are a major cause of lethality and are also responsible for significant morbidity, leading to considerable pain, spinal cord compression and pathological fractures [4].

Metastases are caused by cancer cells disseminated to secondary tissues during different stages of primary tumor progression and often remained dormant for variable periods of time [5,6]. However, metastatic dissemination could take place also *after *primary therapeutic intervention and can be caused by cancer cells departing from either residual tumor or recurrences. For instance, the detection of positive surgical margins upon resection of breast tumors is a common occurrence and is directly related to the incidence of tumor recurrence [1,7,8]. Prior to re-intervention, residual cancer cells in patients with positive resection margins may benefit from a fertile stromal environment that promotes dissemination [9]. This process would produce secondary waves of micrometastases with - at least - equal probability of developing into macroscopic tumors as those seeded years earlier. Thus, the adoption of adjuvant measures aimed to interfere with the arrival of cancer cells to the skeleton would protect breast cancer patients from post-surgery tumor dissemination.

The arrest of circulating cancer cells to the skeleton is highly dependent on specific adhesive interactions with the endothelial cells lining the marrow sinusoids [10-12]. The required next step is the extravasation of adherent cancer cells drawn by chemo attractant cues generated by the surrounding stroma [13]. The similarities between cancer cell dissemination and leukocyte trafficking lead to the identification of chemokines as crucial players in both sets of events [14]. The chemokine CX3CL1 (Fractalkine or FKN, which will be used throughout the rest of the manuscript) exists as a trans-membrane protein that is cleaved into a soluble molecule with potent chemoattractant properties [15]. In its membrane-bound form, FKN can establish strong and stable adhesive interactions with its receptor CX_3_CR1. In contrast to other chemokines, adhesion through FKN does not require activation of additional adhesion molecules via intracellular signaling pathways [16-18]. Because of its unique structural and functional properties, FKN is an ideal candidate to mediate both adhesion and extravasation of CX_3_CR1-bearing circulating cancer cells.

We were the first to report that prostate cancer cells express CX_3_CR1 and that these cells, under dynamic-flow conditions, adhere to human bone marrow endothelial cells in a FKN-dependent manner [19]. In addition, we have shown that CX_3_CR1 is expressed in a high percentage of prostate cancer tissues while human bone marrow supernatants contain soluble FKN, which is released from cells of the bone stroma through a mechanism regulated by androgens [20].

Here we show that both normal and malignant breast tissues express CX_3_CR1 and the ability of breast cancer cells to lodge in the skeleton of animal models is increased by the over-expression of CX_3_CR1. Remarkably, when breast cancer cells were inoculated in transgenic mice knockout for FKN (FKN^-/-^) via a hematogeneous route, a 70% reduction in the detection of bone disseminated tumor cells (DTC) was observed as compared to FKN-expressing animals. Finally, by using functional mutants of CX_3_CR1 we provide evidence that this receptor regulates both adhesion and extravasation of breast cancer cells.

## Materials and methods

### Cell lines and cell cultures

MDA-MB-231 (MDA-231) and MDA-MB-436 (MDA-436) human breast cancer cells were purchased from ATCC (Manassas, VA, USA). All cells were grown in DMEM containing 10% fetal bovine serum (Hyclone, Logan, UT, USA) and 0.1% gentamicin (Invitrogen, Carlsbad, CA, USA) and kept at 37°C and 5% CO_2_. For the experiments performed *in vivo*, cells were engineered to stably express enhanced Green Fluorescent Protein (eGFP) using a lentiviral vector from America Pharma Source (Bethesda, MD, USA). Transduced cells were enriched for eGFP expression by flow cytometry and sorting.

### Transfection and selection of stable cell lines

The cDNAs for wild-type and mutant CX_3_CR1 isoforms were inserted in the pEGFP-N1 vector (Clontech, Inc., Mountain View, CA, USA). MDA-436 cells were transfected with 3 μg of plasmid DNA using the Lipofectamine 2000 transfection system according to the manufacturer's instructions (Invitrogen). Stable transfected cells were selected using geneticin (Invitrogen).

### Immunohistochemistry and tissue array analysis

Three different breast tissue microarrays (BRC1502, BR1002 and BR722) were obtained from US Biomax (Rockville, MD, USA) and included 202 tissue cores of breast cancer and 47 cores of normal breast tissue. The staining for CX_3_CR1 was performed as described previously [20], using an antibody against CX_3_CR1 (7201) obtained from Abcam (Cambridge, MA, USA) and used at a 3.3 μg/ml concentration. Negative controls were obtained by omitting the primary antibody.

### Animal models of metastasis

Five week-old female CB17-SCID, C57Bl/6 and Balb/c mice were obtained from Taconic (Germantown, NY, USA) and housed in a germ-free barrier. C57Bl/6-FKN^-/- ^transgenic mice were obtained from Dr. Sergio Lira (Mount Sinai School of Medicine, NY, USA) and Schering-Plough (now Merck-Schering Plough, Whitehouse Station, NJ, USA) and bred in-house. C57BI/6 mice were used as same-strain controls for the C57Bl/6-FKN^-/- ^transgenic mice to detect cancer cells disseminated to the skeleton at 24 hours post-inoculation. The engrafting of human cancer cells is conventionally conducted using immune-compromised mice and aims to avoid the elimination of the xenogeneic human cells by the immune system of the recipient animal. Interestingly, there were no significant differences in the extent of bone dissemination observed in Balb/C, SCID or C57BI/6 examined at 24 hours post cell-inoculation. This indicates that, within this time frame, the fully competent immune system of Balb/C and C57BI/6 mice is unable to affect the survival of human cells grafted in the blood circulation. However, we considered that the possibility for an acute humoral xenograft rejection (AHXR) between 24 and 72 hours post-inoculation of human cancer cells was substantial. While T- and B-lymphocytes are strongly implicated in the establishment of AHXR [21], SCID mice have fully functional NK cells and macrophages but lack T- and B-lymphocytes [22]. Thus, these animals were used for the experiments measuring the number of bone DTCs at 72 hours post-inoculation.

At six to eight weeks of age, mice were anesthetized with the combined administration of ketamine (80 mg/kg) and xylazine (10 mg/kg) administered by intraperitoneal route and then inoculated in the left cardiac ventricle with either MDA-436 or MDA-231 human cancer cells. Cell inoculation was performed using an insulin syringe with a 30-gauge needle. The correct execution of intracardiac inoculation was established by the appearance of fresh arterial blood in the Luer-Lok fitting of the hypodermic needle, which indicated the successful penetration of the ventricular wall. In addition, blue-fluorescent polystyrene beads (10 μm diameter, Invitrogen-Molecular Probes) were co-injected with cancer cells. Their detection by fluorescence microscopy in different organs at necropsy confirmed the successful inoculation in the blood circulation.

We found that MDA-436 cells are much less effective in disseminating to the skeleton as compared to MDA-231 cells. Thus, for the experiments comparing metastatic dissemination of MDA-436 and MDA-231 cells or in which MDA-436 cells expressing either wild-type CX_3_CR1 or one of its functional mutants were inoculated alone, we used 5 × 10^5 ^cells in a total volume of 200 μl of DMEM/F12. However, for the experiments comparing the number of DTCs in FKN-expressing and FKN(-/-) mice, we inoculated only 1 × 10^5 ^MDA-231 cells.

All experiments were performed in accordance with NIH guidelines for the humane use of animals. All protocols involving the use of animals were approved by the Drexel University College of Medicine Committee for the Use and Care of Animals.

### Tissue preparation and cancer cell detection

Animals were sacrificed and tissues were fixed, decalcified in 0.5 M EDTA if necessary and frozen in O.C.T. embedding medium (Electron Microscopy Sciences, Hatfield, PA, USA) as previously described [23]. Serial tissue sections of 80 μm in thickness were obtained using a Microm HM550 cryostat (Mikron, San Marcos, CA, USA). Sections of each hind leg and soft-tissue organs were transferred on glass slides, stored at -20°C and examined for cancer cells using either an Olympus IX70 fluorescence inverted microscope or an Olympus SZX12 fluorescence stereomicroscope. Bright field and fluorescence images were acquired with an Olympus DT70 CCD color camera.

### Detection of soluble FKN in murine bone marrow

Bone marrow was flushed from the hind legs of wild-type C57Bl/6 mice or FKN-null mice. The cellular fraction was removed by centrifugation at 2,000 r.p.m. for 10 minutes at 4°C. Soluble FKN was detected using an ELISA DuoSet kit for murine FKN (R&D Systems, Minneapolis, MN, USA) as previously described [20].

### CX_3_CR1 signaling *in vitro*

Cells were serum starved for four hours and then exposed to 50 nM recombinant human FKN (R&D Systems) for indicated time points.

### Cell surface CX_3_CR1 protein isolation

The amount of either wild-type or functional mutant forms of CX_3_CR1 that were expressed by MDA-436 breast cancer cells at the plasma membrane level were measured by cell surface biotinylation, using a dedicated kit (cat. #89881) obtained from Pierce (Rockford, IL, USA) and according to the protocol provided by the manufacturer.

### SDS-PAGE and Western blotting

Cell lysates were obtained and SDS-polyacrylamide gel electrophoresis and Western blot analysis were performed as previously described [19], with few modifications. Membranes were probed with an antibody against CX_3_CR1 (0.5 μg/ml, Torrey Pines Biolabs, East Orange, NJ, USA) using 5% milk as a blocking reagent. Membranes were also probed with antibodies targeting phospho-p44/42 MAPK (Thr202/Tyr204, Cell Signaling, Beverly, MA, USA) and total p44/42 MAPK (Cell Signaling). Normalization of gel loading was achieved by using an antibody for beta-actin (Sigma, St. Louis, MO, USA). All primary antibody incubations were performed overnight at 4°C. Primary antibody binding was detected using horseradish peroxidase-conjugated anti-rabbit secondary antibody (Pierce). Chemiluminescent signals were obtained using SuperSignal West Femto reagents (Pierce) and detected with the Fluorochem 8900 imaging system and relative software (Alpha Innotech, San Leandro, CA, USA).

### Statistics

Statistical significance for the *in vivo *studies was determined using a one-tailed Student's *t*-test using GraphPad Prism version 3.0 (GraphPad Software, San Diego, CA, USA) and data are presented as mean ± standard error of the mean (S.E.M.). The significance of CX_3_CR1 staining of TMAs was established by a two-tailed Fisher's exact test performed using GraphPad Prism and using the method of summing small *P*-values.

## Results and discussion

This study was based on the working hypothesis that specific interactions between the receptor CX_3_CR1 and its chemokine ligand FKN are responsible for the arrival and lodging of circulating breast cancer cells to the skeleton.

**Figure 1:**
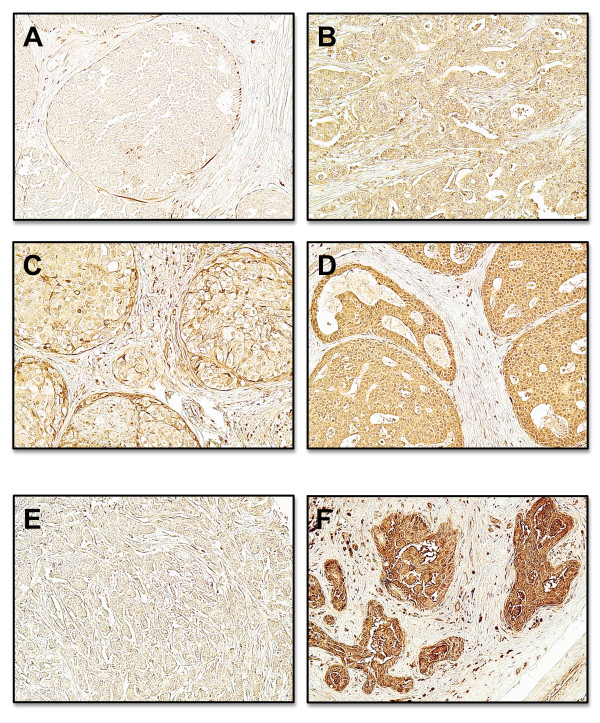
**Expression of CX**_**3**_**CR1 in human breast cancer tissue arrays**. Panel **A **shows a representative sample that stained negative for CX_3_CR1. The majority of samples examined showed different degrees of positive staining for the receptor in the epithelial cells (**B-D; **see also Table 1). Panels **E **and **F **show a negative and highly positive sample for CX_3_CR1, respectively, at higher magnification. The stromal compartment stained uniformly negative for CX_3_CR1. Representative images of 47 normal and 202 malignant tissue cores analyzed. (Original magnification ×100 for A to D and ×200 for E and F).

**Figure 2:**
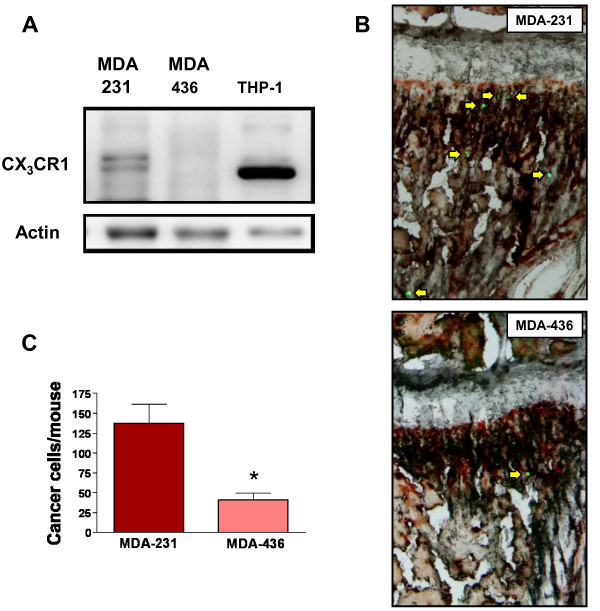
**Expression of CX**_**3**_**CR1 by human breast cancer cells**. Western blotting analysis of CX_3_CR1 expression in MDA-231 and MDA-436 breast cancer cell lines. CX_3_CR1 was detected in MDA-231 cells, whereas MDA-436 showed negligible levels of the receptor. The two bands observed are likely due to the detection of different isoforms of the receptor produced by alternative splicing [33]. Cell lysates from the THP-1 human monocytic cell line were included as a positive control. Actin was used as a loading control (**A**); single breast cancer cells stably expressing eGFP (arrows) were identified by fluorescence stereomicroscopy in the bone marrow of mice, 24 hours after being inoculated *via *the left cardiac ventricle (**B**); MDA-231 and MDA-436 cell lines that migrated to the femora and tibiae of mice inoculated via the hematogenous route, expressed as mean of cells detected per animal + S.E.M. (**C**). (MDA-231 cells = four mice, MDA-436 cells = eight mice. * *P *= 0.0008).

**Figure 3:**
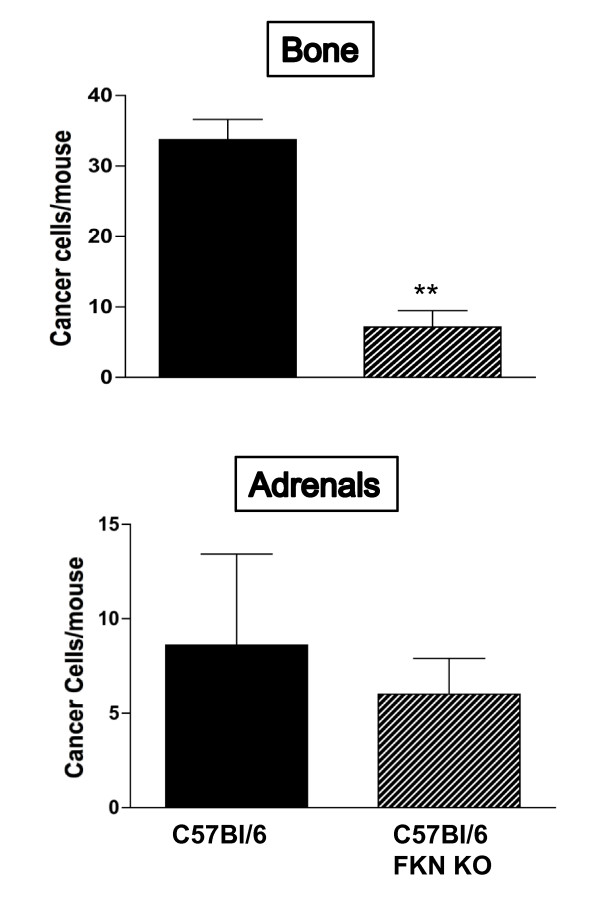
**Reduced homing of CX**_**3**_**CR1-expressing cancer cells in the bone of FKN-null mice**. The number of MDA-231 cells detected in the skeleton of transgenic FKN(-/-) mice (*n *= 5) 24 hours after intra-cardiac inoculation is significantly decreased compared to the number of cells detected in wild-type C56BI/6 mice (*n *= 4). The number of cells detected in the femora and tibiae per animal is shown as mean + S.E.M. (**-*P *= 0.0002).

**Figure 4:**
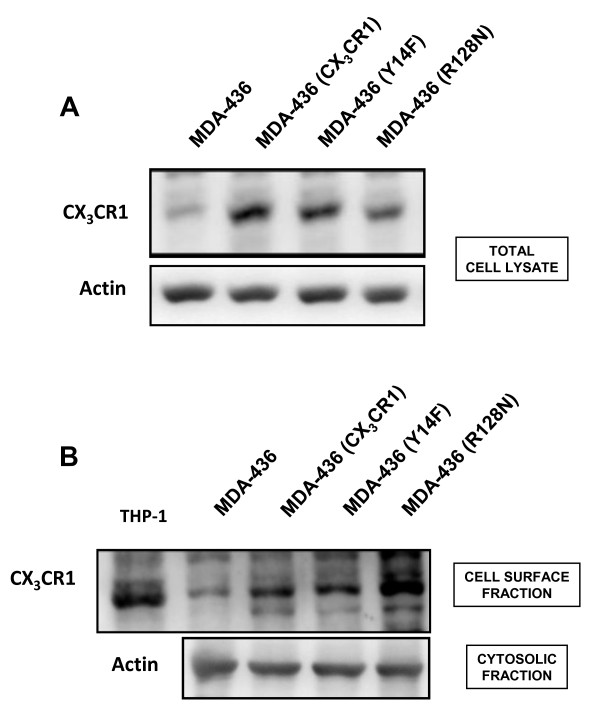
**Exogenous expression of wild-type and functional mutants of the CX**_**3**_**CR1 receptor in MDA-436 breast cancer cells**. Western blotting analysis of total cell lysates collected from parental MDA-436 cells as well as cells stably overexpressing the wild-type CX_3_CR1, the adhesion-impaired Y14F mutant or the chemotaxis-impaired R128N mutant of the receptor. Actin was used as a loading control (**A**); the correct insertion of each exogenously overexpressed form of the CX_3_CR1 receptor at the plasma membrane level of transfected MDA-436 cells was confirmed by cell-surface protein isolation (**B**). Cell lysates from the THP-1 human monocytic cell line were included as a positive control for CX_3_CR1 expression. Actin detected in the cytosolic fraction was used as a loading control.

To ascertain whether human breast expresses the chemokine receptor, we processed human tissue microarrays of normal and malignant mammary glands for CX_3_CR1 detection by immunohistochemistry. As shown by Figure 1, both normal and tumor tissues stained diffusely for CX_3_CR1 and the signal was strictly limited to the epithelial compartment. However, 26% of malignant mammary glands were characterized by the strongest staining intensity observed and broad signal distribution: only 1 out of 47 samples of normal mammary tissue demonstrated this similar staining pattern (Table 1). Thus, the CX_3_CR1 chemokine receptor is frequently detected in normal mammary glands where its expression and distribution increases with malignant transformation. This scenario closely resembles what we previously described for human prostate tissue [20]. Our previous studies with human prostate cancer cell lines also revealed that the CX_3_CR1-FKN pair mediates both the adhesion of cancer cells to endothelial cells of the bone marrow under dynamic-flow conditions and their migration following chemoattractant gradients *in vitro *[19]. These events are crucial to ensure the arrest and lodging of cancer cells departing from the primary tumor and arriving to the skeleton through the hematogenous route. Because of the strong propensity to disseminate and grow in the bone shown by breast adenocarcinoma, we asked whether a correlation could be found between the levels of CX_3_CR1 expression and bone-metastatic potential of human breast cancer cell lines. Thus, we selected MDA-231 cells, which are widely recognized as strongly bone-metastatic [24-26], and MDA-436 cells for which the ability to effectively target the skeleton and grow into macroscopic metastases in animal models has never been reported. When these two cell lines were tested for CX_3_CR1 by Western blotting, MDA-231 cells were found positive whereas MDA-436 express minimal levels of this receptor (Figure 2A). To investigate whether this disparity in CX_3_CR1 expression could account for a different ability to arrest at the skeleton, we tested these two breast cancer cell lines by delivering them into the blood circulation of mice. As previously described, we have established an animal model of metastatic dissemination that allows the identification of single cancer cells immediately after their arrival to different tissues, including the bone [23]. We have also shown that prostate cancer cells delivered into the arterial blood circulation consistently lodge at the metaphysis of the distal femur and proximal tibia of inoculated animals [23,27-29]. Using a similar approach for this study, mice were inoculated via the left cardiac ventricle with MDA-231 or MD-436 cells that stably expressed eGFP and sacrificed 24 hours later. Tibiae and femora of each mouse were collected, processed for cryosectioning and found to be harboring cancer cells when inspected by fluorescence microscopy. DTC were identified and counted throughout each entire bone by analyzing all serial sections obtained. These experiments revealed a much stronger propensity of MDA-231 cells to home to the bone marrow than MDA-436 cells (Figure 2B, C). The three-fold higher number of MDA-231 cells detected in the bone of inoculated mice correlates with the higher expression of CX_3_CR1 detected in these cells as compared to MDA-436 cells. However, more causal evidence for the role exerted by the CX_3_CR1-FKN pair in breast cancer cell homing to the bone would require interfering with the molecular interactions between the chemokine and its receptor *in vivo*. This opportunity was offered by a FKN-null transgenic mouse previously generated by Dr. Lira and collaborators [30]. So far, this approach could not be pursued to test the role in metastasis of other chemokines, such as CXCL12/SDF1, since CXCL12-null transgenic mice are not viable. In contrast, besides surviving targeted gene-disruption, FKN-null mice do not exhibit overt behavioral abnormalities or macroscopic anatomical alterations. The CX_3_CR1-FKN pair is involved in leukocyte trafficking and immune response in general; an in-depth analysis of these animals revealed that the responses to inflammatory stimuli were comparable to those of C57Bl/6 wild-type mice. We used an ELISA-based assay and confirmed the absence of the soluble chemokine in the bone marrow of FKN(-/-) mice, which, in contrast, was detected in the parental C57Bl/6 mouse strain at an average concentration of 6.4 ng/ml. FKN(-/-) mice inoculated with CX_3_CR1-expressing MDA-231 cells displayed a dramatic reduction of more than 70% in the number of breast DTC detected 24 hours later in the bone marrow of tibia and femur, as compared to wild-type animals (Figure 3). As a small number of DTC were still detected in the bone of FKN(-/-) mice, it is plausible that additional molecules are implicated in the homing of these breast cancer cells to the skeleton. However, the dramatic reduction of bone DTC upon ablation of FKN indicates that CX_3_CR1 expression should be considered an important feature of breast cancer cells with bone-metastatic potential. Interestingly, we observed no differences between wild-type and FKN(-/-) mice in the number of MDA-231 cells detected in the adrenal glands, which are soft-tissue organs that can be also moderately colonized in our animal model (Figure 4). This rules out the possibility that the targeted ablation of FKN could non-specifically affect cell dissemination through blood circulation and reinforces the specificity of the CX_3_CR1-FKN pair in regulating the migration of cancer cell to the skeleton.

**Table 1:** Intensity and distribution of CX_3_CR1 in normal and malignant breast tissues

Tissues	0	0 to 1	1 to 2	2 to 3	Total
Normal	21 (45%)	19 (40%)	6 (13%)	1 (2%)	47
Malignant	43 (21%)	54 (27%)	53 (26%)	52 (26%)	202

Staining intensity was scored based on a scale in which negative specimens were graded (0) and strongly positive samples were graded (3). To evaluate distribution, tissues with complete lack of staining were scored as (0); tissues prevalently negative for CX_3_CR1 displaying some areas of positive staining were scored as (0 to 1); tissues showing uniform staining for CX_3_CR1 were scored as either (1 to 2) or (2 to 3), based on the intensity of the signal observed. The increase in CX_3_CR1 expression observed in cancer samples as compared to normal samples resulted to be statistically significant with a *P-*value equal to 0.0016.

In light of the *in vivo *results provided by the FKN(-/-) mouse model, we decided to further investigate the involvement of CX_3_CR1 in breast cancer metastasis by exogenously expressing this receptor in MDA-436 cells, as we found that these cells do not express CX_3_CR1 and migrate to the bone with very low efficacy (Figure 2). In addition, we sought to further dissect the relative impact that CX_3_CR1 exerts in adhesion to the endothelium of bone sinusoids and extravasation into the surrounding bone marrow stroma, respectively. We have previously reported that both adhesion and migration of prostate cancer cells can be regulated by FKN *in vitro *[19]. However, the targeted deletion of this chemokine eliminates both the trans-membrane adhesive molecule and the soluble chemoattractant form, and therefore the FKN(-/-) mice could not be used to address this specific issue. The next series of experiments were, therefore, conducted with MDA-436 cells stably expressing either the wild-type form of CX_3_CR1 or one of the two following functional mutants of this receptor. The first mutant was generated by introducing a tyrosine to phenylalanine mutation at amino acid 14 of the first extracellular domain of CX_3_CR1 (Y14F) [31]. This mutant was previously characterized for its failure to firmly bind to FKN, most likely because of the inability of phenylalanine to be sulfated, a modification that enhances the binding to this chemokine. Although defective in capture and adhesion, CX_3_CR1 (Y14F) is competent in signal transduction, but with a 100-fold decreased affinity to immobilized FKN [31]. The specific involvement of CX_3_CR1 in extravasation was evaluated using a second functional mutant containing an arginine to asparagine mutation at amino acid 128, which is located in the second intracellular loop of CX_3_CR1 and in the highly conserved aspartic acid-arginine-tyrosine (DRY) sequence of G-protein coupled receptors [16]. Chemoattractant properties of chemokine receptors are dependent on G-protein activation and subsequent ability to transduce downstream signals following stimulation by the appropriate ligand [32]. As the *DRY *sequence is required for G-protein activation, the R-to-N mutation makes the receptor incompetent of intracellular signaling [16] and cells expressing the CX_3_CR1 (R128N) mutant do not migrate toward FKN, while showing normal binding/adhesion to this chemokine [16]. The expression of wild-type and mutated forms of CX_3_CR1 by MDA-436 cells was verified by western blotting performed on total cell lysates (Figure 4A).

In addition, the insertion of each form of the receptor at the plasma membrane level of transfected cells was confirmed using Western blot analysis of cell surface proteins isolated by biotinylation (Figure 4B).

**Figure 5:**
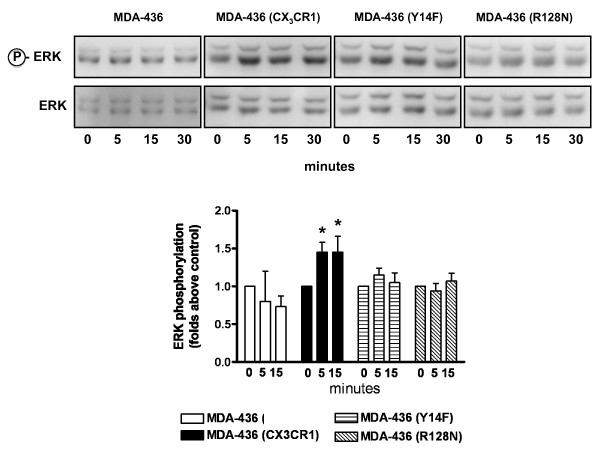
**Intracellular signaling of CX**_**3**_**CR1 and functional mutants exogenously expressed in MDA-436 cells**. Western blotting analysis of MDA-436 cells that when exposed to 50 nM of human FKN failed to phosphorylate Erk1/2, as expected from the minimal expression of endogenous CX_3_CR1. Cells that were engineered to stably overexpress wild-type CX_3_CR1 (MDA-436+X) showed a significant and time-dependent Erk phosphorylation in response to FKN. Finally, cells expressing the Y14F (MDA436+Y14F) or R128N (MDA436+R128N) functional mutants of the receptor showed a negligible or lack of Erk phosphorylation, respectively; the intensity levels of Erk phosphorylation obtained from three independent experiments were evaluated by densitometry analysis and normalized using total Erk signals after membrane stripping to compensate for variations in protein loading among samples. Data are represented as mean ± S.E.M. (* *P *< 0.003).

**Figure 6:**
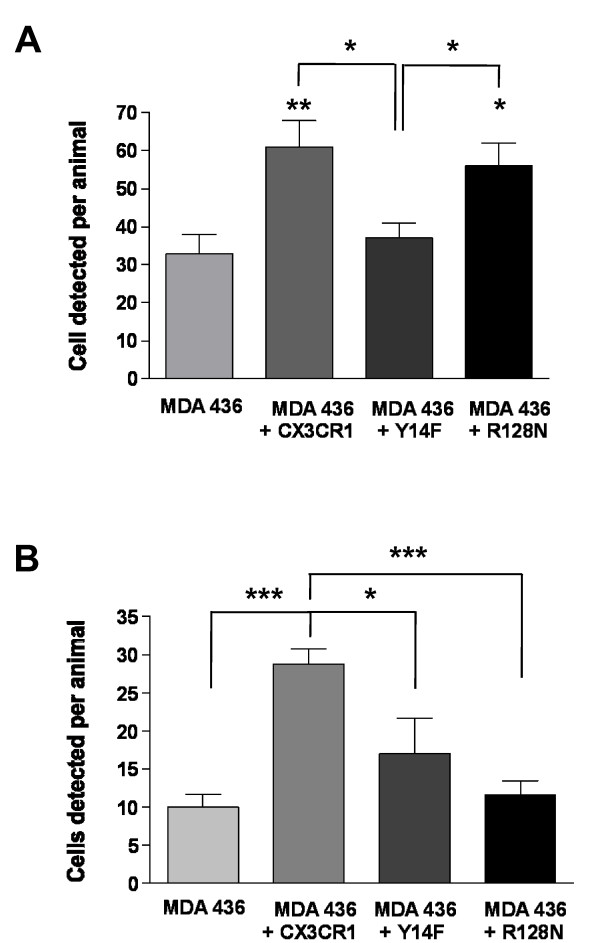
**Role of CX**_**3**_**CR1 in the adhesion and extravasation of breast cancer cells**. The number of MDA-436 cells expressing exogenous CX_3_CR1 that were detected in the femora and tibiae of mice 24 hours after intracardiac inoculation was significantly larger than that of untransfected MDA-436 cells. The expression of the adhesion-defective Y14F mutant of the receptor failed to increase the arrival of MDA-436 cells to the skeleton, whereas the chemotaxis-defective R128N mutant was still able to promote arrival to bone similarly to the wild-type CX_3_CR1 (**A**); when animals were inspected at 72 hours post-inoculation, the difference in bone arrival between parental and CX_3_CR1-expressing MDA-436 cells was even more significant than what was observed after 24 hours. Also, at this later time point, cells expressing the Y14F adhesion-defective mutant were detected in numbers similar to the untrasfected cells. Finally, the cells expressing the R128N mutant, despite their ability to adhere normally to the bone marrow endothelium at 24 hours, failed to extravasate and were then detected in numbers similar to the untransfected cells (**B**). Between four and seven mice were used for the analysis of parental and different CX_3_CR1-expressing MDA-436 cells migrated to the skeleton at each time point. Data are reported as mean ± S.E.M. (*-*P *≤ 0.03, **-*P *≤ 0.006, ***-*P *< 0.0001).

When MDA-436 cells engineered to exogenously express CX_3_CR1 were exposed to 50 nM soluble FKN *in vitro*, a time-dependent phosphorylation of ERK1/2 was observed. As expected, this downstream signaling was not detected in untransfected cells, as they express minimal levels of CX_3_CR1 (Figure 5). The MDA-436 cells expressing the Y14F mutant responded to soluble FKN with a negligible and non-statistically significant activation of the MAPK pathway, most likely as a consequence of the reduced affinity for the chemokine, as discussed above. Finally, the R128N mutant did not transduce downstream signals in MDA-436 cells, confirming its inability to activate G-protein (Figure 5). The role of wild -type and functional mutants of CX_3_CR1 in regulating the homing of breast cancer cells to the skeleton was then tested by inoculating MDA-436 cells in mice as untransfected cells or expressing one of the three available forms of the receptor. Animals were sacrificed at either 24 or 72 hours following inoculation, to allow discriminating between the fast adhesion to the bone marrow endothelium and the relatively delayed extravasation into the surrounding stroma [23]. After 24 hours, the expression of wild-type CX_3_CR1 doubled the number of MDA-436 cells detected in the bones of inoculated mice (Figure 6A). This result provides strong support for the role of CX_3_CR1 in mediating the arrival of circulating breast cancer cells to the skeleton and further validates the significantly higher ability for skeletal dissemination shown by CX_3_CR1-expressing MDA-231 cells as compared to CX_3_CR1-negative MDA-436 cells. Cells expressing the CX_3_CR1(Y14F) mutant arrested to the bone in numbers comparable to cells lacking the receptor, a clear expression of their inability to bind and adhere to FKN on the luminal side of bone marrow endothelial cells. On the other hand, MDA-436 cells expressing the CX_3_CR1(R128N) mutant arrested to the skeleton with an efficiency comparable to cells expressing the wild-type receptor, indicating that extravasation into the marrow stroma required longer than 24 hours from the entering of the blood circulation (Figure 6A). When animals were examined at 72 hours following cell inoculation, the number of MDA-436 cells either lacking or expressing the CX_3_CR1 receptor that were detected in the bone was lower than that observed at 24 hours post-inoculation (Figure 6B). This suggests that a significant number of adherent cells fail to extravasate and are eventually dislodged from the endothelial wall. Interestingly, even in these conditions, CX_3_CR1 still provided MDA-436 cells with a three-fold higher propensity to remain at the skeleton as compared to cells lacking this receptor. The cells expressing either CX_3_CR1(Y14F) or (R128N) mutants failed to show increased lodging in the bone as compared to cells lacking CX_3_CR1 expression. This was plausibly due to their inability to initially adhere to the endothelium or migrating toward the soluble FKN produced and released in the bone marrow by stromal cells [20], respectively.

Taken together, these results provide definitive support to the idea that the interactions between CX_3_CR1 and FKN promote skeletal dissemination of circulating breast cancer cells. Importantly, this observation might be likely extended to other malignant phenotypes, including those deriving from tumors of the prostate gland [19]. In addition, the role exerted by CX_3_CR1 in the arrest to the bone includes the regulation of both cell adhesion and extravasation, in line with the ability of this receptor to interact with both plasma membrane-bound and soluble FKN.

## Conclusions

Based on our study, it seems reasonable to propose that the dissemination to the skeleton of breast cancer cells can be effectively counteracted by interfering with the molecular and functional interactions between the chemokine FKN and its only receptor CX_3_CR1. The detection of this receptor in the majority of human breast tissue samples we examined (Table 1) suggests that this type of strategy could protect a relevant number of patients from skeletal metastases. In light of the concrete possibility of post-surgery spreading, these results should bolster the synthesis of CX_3_CR1 inhibitors and their pre-clinical and clinical testing, to ultimately conceive new adjuvant therapeutic approaches and promote a paradigm shift in the management of breast cancer patients.

## Abbreviations

AHXR: acute humoral xenograft rejection; DTC: disseminated tumor cells; ELISA: enzyme-linked immune assay; FKN: fractalkine; GFP: green fluorescent protein; OCT: optimal cutting temperature; SCID: severe combined immunodeficiency; TMA: tissue microarrays.

## Competing interests

The authors declare that they have no competing interests.

## Authors' contributions

WLJ-G carried out the experiments with the animal models of metastasis, performed the CX_3_CR1 and the immunohistochemistry on TMAs, and helped to draft the manuscript. YZ carried out the cell-surface biotinylation experiments and the signaling studies for wild-type and CX_3_CR1 functional mutants' expression. AMF provided the Y14F and R128N mutants of CX_3_CR1. OM participated in conceiving the study and drafting the manuscript. AF conceived the study, participated in its design and coordination, drafted the manuscript, and scored the TMAs for CX_3_CR1 expression. All authors read and approved the final manuscript.
